# Improving gelatin‐based emulsion films with cold plasma using different gases

**DOI:** 10.1002/fsn3.1939

**Published:** 2020-10-18

**Authors:** Shahrbanoo Ahmadi Ledari, Jafar Mohammadzadeh Milani, Farshad Sohbatzadeh Lanbar

**Affiliations:** ^1^ Department of Food Science and Technology Sari Agricultural Science and Natural Resources University Sari Iran; ^2^ Department of Atomic and Molecular Physics Faculty of Basic Science University of Mazandaran Babolsar Iran

**Keywords:** cold plasma, emulsion films, gelatin, mechanical properties, surface hydrophobicity

## Abstract

In this research, the effects of cold plasma treatment on the properties of gelatin‐based emulsion films (GEFs) using different gases were investigated. The gases used include O_2_, N_2_, air, Ar, and ethanol‐argon (EtOH‐Ar). Surface hydrophobicity, morphology, water vapor permeability (WVP), and mechanical, thermal, and antifungal properties after plasma application on the film were analyzed. The results revealed that surface hydrophilicity significantly increased after cold plasma, while the contact angle significantly decreased (*p* < .05). Furthermore, atomic force microscopy results showed that the argon gas plasma significantly increased roughness of the GEFs surface. Besides, plasma did not decrease WVP. Different gases had no significant effect on the mechanical properties of the GEFs (*p* > .05). Oxygen permeability after plasma application was significantly different from the control sample; consequently, the permeability after plasma application decreased and the lowest level 55.7 (cm^3^μm m^−2^ day^−1^ Pa^−1^) was seen for oxygen gas. Plasma treatment caused etching effects and lessened the surface moisture of the polymer film. Antimicrobial activity was observed in the cold plasma‐treated samples, especially under air and nitrogen atmosphere. Cold plasma treatment is an effective method for surface modification, expanding the application of emulsion films in the packing industry with improved performance properties.

## Graphical abstract

The effects of cold plasma treatment on the properties of the gelatin‐based emulsion films (GEFs) using different gases including O_2_, N_2_, air, Ar, and ethanol‐argon (EtOH‐Ar) were studied via analyzing surface hydrophobicity, morphology, water vapor permeability (WVP), and mechanical, thermal, and antifungal properties.

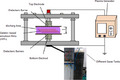

## 1 INTRODUCTION

Common packaging systems have the ability to delay the loss or adsorption of moisture between the food and the surrounding environment, but they cannot prevent the transport of moisture, fats, and other compounds inside the food package. Edible films and coatings can solve this problem. In addition, the addition of active compounds prevent spoilage of food, maintaining its quality during transportation and storage, and after decomposition, they are degraded to water, carbon dioxide, and inorganic compound in a short time without any toxic residue and environmental problems (Sobral et al., 2001).

Currently, food packaging researches have mostly focused on biodegradable edible films and using of animal and vegetable protein sources (Tharanathan, 2003). The edible films made of proteins have good mechanical properties but high moisture permeability conversely, the films from lipid compounds have low water vapor permeability (WVP) but poor mechanical properties (Perez‐Gago & Krochta, 2001). By mixing these two types of materials, the resulting composite films will have the desired properties.

Composite films are made as laminated or emulsions films. Although the laminated composites films have better resistance to water vapor, the emulsion films are preferred, due to the difficulty of the preparation process. Numerous studies have reported that vegetable oils can be used to incorporate lipid into the matrix of biopolymers (Galus, 2018; Galus & Kadzińska, 2015; Pereda et al., 2014; Sahraee, et al., 2017; Sahraee, et al., 2017; Valenzuela et al., 2013). Vegetable oils (olive, sunflower, corn, rapeseed) are readily accessible, inexpensive, nontoxic, unlimited, and nonvolatile; besides, they are the source of unsaturated fatty acids and mixing them, in the form of coating, with edible food products have various health benefits (Galus & Kadzińska, 2015).

In most cases, different surface treatments are used to obtain polymers with desirable properties. Cold plasma involves several physical and chemical processes that alter the surface properties of the polymer in plasma volume (Poncin‐Epaillard et al., 1999). The term plasma refers to quasi‐neutralized ionized gas, mainly consists of photons, ions, free electrons, and atoms in the normal or excited state with a neutral charge. It is considered to be the fourth state of the fundamental material and could be classified as thermal or nonthermal plasma, depending on its density and electronic temperature (Zhu et al., 2011).

Cold plasma is a new technology that offers many possible usages for food packaging. While cold plasma was initially industrialized to raise the energy levels of polymers and increase their adhesion and printing, it has lately appeared as a powerful tool for surface sterilization of food and food packing materials (Pankaj et al., 2015). Cold plasma processing can significantly reduce WVP due to increased twisting in the diffusion path (Dong et al., 2018). Plasma gaseous reactions can inactivate microorganisms adhered to the surfaces of the polymer over a short period. Packaging materials including plastic bottles, caps, and films can be quickly sterilized using cold plasma, without affecting the general properties or leaving any residues (Muranyi et al., 2010).

Air, O_2_, He, N_2_, and Ar gases are often used for surface modification of polymers. N_2_ and O_2_ are used for degradation and surface functionalization of polymer, while helium and argon cause negligible chemical effects and are used in etching (Oh et al., 2016). Therefore, understanding the plasma treatment effects using the mentioned gases is important to determine the optimum circumstances for cold plasma treatment (Almazán‐Almazán et al., 2006). Numerous studies have concentrated on improving the kinetic physical properties of biodegradable polymers using cold plasma (Dong et al., 2017; Oh et al., 2016; Pandiyaraj et al., 2015; Romani et al., 2019; Song et al., 2016).

The aim of this study is to investigate the effect of cold plasma treatment using different gases on gelatin‐based emulsion films (GEFs) properties such as water vapor and oxygen permeability, mechanical and antimicrobial properties of the gelatin‐corn oil emulsion films.

## 2 MATERIALS AND METHODS

### 2.1 Materials

Gelatin powder was purchased from Samchun Pure Chemicals Co. (Korea). Corn oil was obtained from Varamin Co. (Rudbar, Iran). Tween (822187) and 50% Glutaraldehyde (814393) was obtained from Merck Co. (Germany). *Aspergillus niger* mold (PTTCC: 5010) was obtained from Iran Scientific‐Industrial Research Center.

### 2.2 Preparation of GEFs

**Figure 1:**
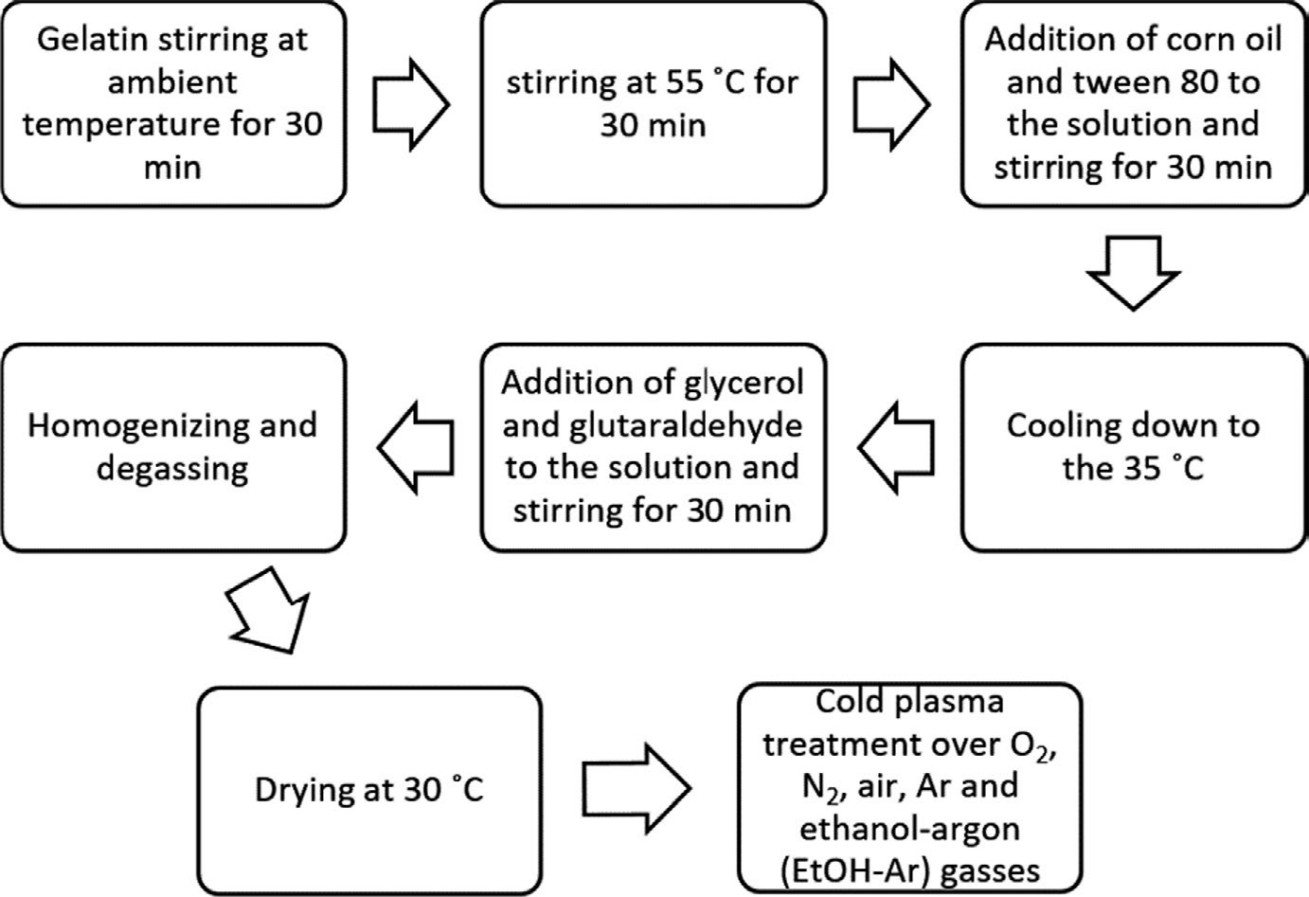
Schematic diagram of gelatin emulsion film preparation

The procedure of GEFs preparation has been shown in Figure 1. Gelatin (4 g per 100 ml) was stirred (MS300HS) at room temperature for 30 min followed by another 30 min at 55°C. Then, corn oil with a 7.5% concentration based on dry gelatin (Seyedi et al., 2015) and Tween‐80 was added to as an emulsifier the solution and mixed for 30 min at 55°C with a magnetic stirrer. After the mixture was cooled down to 35°C, 30% glycerol (gelatin‐based dry matter), as a plasticizer, and 1% glutaraldehyde were added as cross‐linking agents to the solution and stirred for another 30 min. The resulted solution was homogenized and degassed by a vacuum pump. Finally, the film solution was poured onto Teflon dishes and dried in an oven at 30°C (Nafchi et al., 2014). After drying, the films were exposed to plasma treatment by various gases.

### 2.3 Cold plasma treatment of GEFs

**Figure 2:**
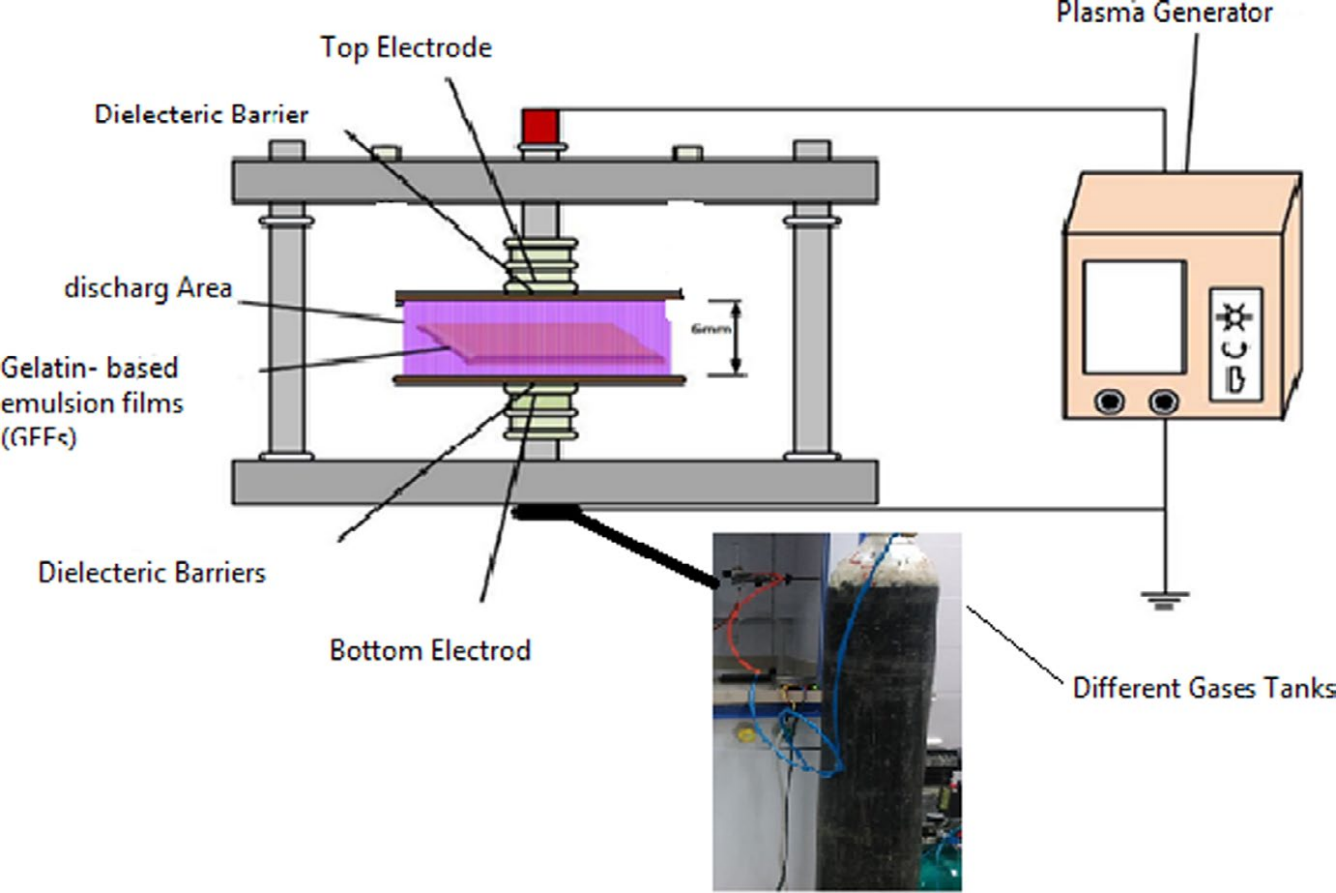
Schematic of equipment for plasma system

The cold plasma (dielectric barrier discharge) setup was comprised of two 6 × 16 cm aluminum electrodes with a 6 mm gap, each of which was coated with a layer of polytetrafluoroethylene with a thickness of 1 mm. The two ends of the electrode were connected to an AC voltage source with a frequency of 375 Hz. The film dimensions for plasma application were 14 × 4 cm. Plasma was used at atmospheric pressure by air, nitrogen, oxygen, argon, and argon ethanol gases. Plasma was applied to the film samples for 15 min with air powers of 19, argon 16.5, nitrogen 30, oxygen 27, and ethanol‐argon 19.5 W. Since the breakdown of different gases is different, our power was also different. The schematic of plasma equipment is shown in Figure 2.

### 2.4 GEFs characterization

#### 2.4.1 Mechanical properties

Tensile properties of GEFs, including elongation to breakpoint (EB), tensile strength (TS), and Young's modulus (YM), were measured according to ASTM standard (2000) using a Brookfield CT3 device. The films were cut into 1 × 10 cm pieces and conditioned at a relative humidity of 52% and a temperature of 23°C for 48 hr before the test (Sahraee, et al., 2017; Sahraee, et al., 2017).

#### 2.4.2 Water vapor permeability

Water vapor permeability of the films was measured according to ASTM E96‐00 standard method. In this method, glass vials with 1.5 cm diameter and 4 cm depth were filled with silica gel to reduce their internal moisture to 0%. The film specimens were fixed into a vial with a 4 mm diameter hole. After measuring the initial weight, the vials were placed in a desiccator containing a saturated solution of K_2_SO_4_ to provide a relative humidity of 97% outside the vials. The weight of the vials was measured in regular intervals (24 hr) for one week, and the water vapor transfer rate (WVTR) was determined from the slope of the linear chart of the film weight versus time. Water vapor permeability of the films (g s^−1^ m^−1^ Pa^−1^) can be acquired using the following equation:

(1) $$\text{WVTR}=\frac{\text{slope}}{A}$$

(2) WVP=(WVTR×L)/ΔP

where *L* is the average film thickness, *A* is vial opening area in square meters, and Δ*P* is the difference of vapor pressure between the inside and outside of the vials.

#### 2.4.3 Contact angle

In order to study the effect of plasma on GEFs surface hydrophobicity, the films were kept in the relative humidity of 52% ± 2% and 23 ± 2°C for 2 days, and the contact angle was measured. For this test, square pieces of GEFs (2 × 2 cm^2^) were located on the black surface. Then, 10 μl of water drop were poured onto the film surface from a specified distance (10 mm) and contact angle was recorded immediately. Images from different angles were taken by a digital camera (Dino Capture Lite) (Liu et al., 2015). For each film, at least three replicates were tested.

#### 2.4.4 Differential scanning calorimetry (DSC)

The thermal properties of the films were analyzed by a differential scanning calorimeter (DSC‐model S.DS.T‐400‐IRAN). 15 mg of the films were heated under a nitrogen atmosphere at a thermal range of 30–350°C at a speed of 10°C/min (De Carvalho & Grosso, 2004).

#### 2.4.5 Atomic force microscopy (AFM)

The surface of GEFs before and after plasma treatment was observed by atomic force microscopy. A double‐sided tape was used to fix the film. Tapping mode was applied to the microscope to measure the surface topographic changes with changes in the amplitude of the tip contact at the sample surface. Data analysis was performed using Imager (version 1.00 Aras Research Co.) software (Ara Research Company Code: AI4‐024/0).

#### 2.4.6 Fourier transform infrared spectroscopy (FTIR)

Fourier transform infrared spectroscopy (Cary 630, 2017, USA) (FTIR) was used to collect the infrared spectra. The spectra of the samples were obtained at frequencies between 400 and 4,000 cm^−1^, a resolution of 4 cm^−1^, and a scanning rate of 16. The obtained spectra were then analyzed to determine the types of possible links and functional groups. In this test, potassium bromide was used as a reference (Maran et al., 2013).

#### 2.4.7 Antimicrobial activity

Antimicrobial activity was evaluated by disk diffusion assay. Potato dextrose agar (PDA) medium was tipped into sterile Petri dishes and frozen. The suspension of *Aspergillus niger* (10^6^ cfu/ml) was cultured on this medium. Circles with a diameter of 16 mm were cut by round punches and transferred to the surface of the medium. The plates were incubated at 4°C for 24 hr and then examined for the inhibition zones. Inhibition zones were measured as the diameter of nongrowth zones including a polymer disk diameter at the end of incubation. Then, using a digital Vernier caliper, the growth zone diameter (mm) was determined as an indicator of antimicrobial activity. An empty plate of nutrient agar was considered as the control sample (Sanuja et al., 2015).

#### 2.4.8 Oxygen permeability

The oxygen permeability was assessed according to the ASTM (2000) reference method. A sample film was placed between two chambers with two channels, one for gas inlet and the other for gas outlet. In the lower chamber, O_2_ flowed at a controlled rate and an electronic flow meter was used to keep the pressure constant in this section. The other chamber was filled with nitrogen as an O_2_ carrier, which was also controlled. O_2_ penetration values (cm^3^μm m^−2^ day^−1^ Pa^−1^) were determined by gas chromatography (Ramos et al., 2013).

### 2.5 Statistical analysis

A completely randomized design was used to compare different treatments. One‐way analysis of variance (ANOVA) was used in SAS.9.4 software. Duncan's test was used to determine the significant difference (*p* < .05) between means and the final values were reported in the form of mean ± standard deviation. All experiments were performed in triplicate.

## 3 RESULTS AND DISCUSSION

### 3.1 Mechanical properties

The mechanical properties of the cold plasma‐treated samples are shown in Table 1. Different gases had no considerable effect on the mechanical properties of the GEFs (*p* > .05). Mechanical properties are one of the most important properties of food packing materials. Optimizing the mechanical properties of edible films is important in several respects, such as high mechanical strength of the film which causes mechanical damage such as piercing and consequently its deterrent property due to stress on the packaging material, to protect against gases and moisture. The high flexibility of the film makes it easy to adapt to the shape of the food without being fractured and easily used as a coating. The film's high resistance also protects the nutrient inside it from stress during transport. Films made from natural polymers generally exhibit poor functional properties compared with synthetic types. But among natural polymers, proteins show better mechanical properties than polysaccharide and lipid films due to their unique structure based on 20 different amino acids (and multiple intermolecular bonds in their structure) (Zhou et al., 2009).

**Table 1:** Effect of cold plasma treatment using different gases on the mechanical properties of GEFs

Plasma gases	Thickness (mm)	SB	TS (MPa)	EB (%)
Air	0.126 ± 0.01^a^	0.48103 ± 0.06^a^	1,935.3 ± 88^a^	684.4 ± 5.2^b^
O_2_	0.125 ± 0.01^a^	0.63817 ± 0.09^a^	1,855.3 ± 80^a^	985.1 ± 5.8^ab^
N_2_	0.146 ± 0.03^a^	0.65600 ± 0.12^a^	1,430 ± 74^a^	561.5 ± 4.8^b^
Ar	0.144 ± 0.01^a^	0.60037 ± 0.13^a^	1,653 ± 62^a^	747.2 ± 4.3^b^
EtOH‐Ar	0.126 ± 0.02^a^	0.62183 ± 0.11^a^	1,675.3 ± 55^a^	675.3 ± 5.4^a^
Control	0.131 ± 0.02^a^	0.62853 ± 0.11^a^	1,986.3 ± 63^a^	816.2 ± 5.3^ab^

Note

Data are reported as mean ± *SD*. Means with superscripts of different letters are significantly different (*p* < .05).

Abbreviations: EB, elongation to break; SB, strain to break; TS, tensile strength.

Tensile strength (TS), Elongation to Break (EB), Strain to Break (SB), and elastic modulus (EM) are important parameters of films that can be used as an indicator of film to maintain film integrity and resistance to environmental stresses during packaging. Our results indicate that plasma treatment alone cannot improve the mechanical properties of the film (*p* > .05).

### 3.2 Water vapor permeability

The WVP property of packaging materials is an important factor in determining the shelf life of food products. The WVP data are shown in Table 2. Plasma treatment with different gases showed no significant difference in inhibitory properties of the films against water vapor permeation (*p* > .05) compared with the control sample, which was 1.68 (g s^−1^ m^−1^ Pa^−1^). The effects of plasma on moisture deterrence properties of food packaging polymers have been studied in this field, because inhibitory properties are important factors in controlling the shelf life of foods (Pankaj et al., 2014).

**Table 2:** Water vapor permeability (WVP), contact angle, and oxygen permeability (OP) of GEFs

Plasma gases	WVP (×10^−10^ g^−1^ s^−1^ Pa^−1^)	Contact angle (°)	OP (cm3μm m^−2^day^−1^Pa^−1^)
Air	1.72 ± 0. 08^a^	40.7 ± 0.3^c^	247.5^b^
O_2_	1.78 ± 0. 06^a^	44.5 ± 0.3^b^	55.7^f^
N_2_	1.79 ± 0. 05^a^	45.3 ± 0.5^b^	179.5^c^
Ar	1.69 ± 0. 05^a^	36.7 ± 0.4^d^	102.3^e^
EtOH‐Ar	1.69 ± 0. 05^a^	34.6 ± 0.6^e^	113.4^d^
Control	1.68 ± 0. 06^a^	57.1 ± 0.6^a^	326.6^a^

Note

Data are reported as mean ± *SD*. Means with superscripts of different letters are significantly different (*p* < .05).

Plasma treatment with oxygen gas formed functional groups containing oxygen such as peroxide, carboxylic acid, and hydroxyl, while plasma treatment with nitrogen gas formed C–N, C = N, and amide bonds at the surface of the polymer (Morent et al., 2011). Unlike O_2_ permeability, the penetration of the polar water vapor molecule did not mainly depend on the opening of the polymer chains. Therefore, WVP is not reduced by more difficult chain opening of plasma‐treated polymers, caused by higher adhesion energy density. A water vapor molecule using hydrogen bonding could diffuse through the film grid even if the cohesive energy density of the films was relatively high (Moosavi et al., 2019). The results indicated that the treatment of the films with cold plasma using different gases did not change their WVP.

### 3.3 Oxygen permeability

The oxygen permeability test results are illustrated in Table 2. As shown, the results were significantly different for different plasma gases (*p* < .05). All plasma‐treated samples showed significantly lower oxygen permeability compared with the control sample.

The oxygen permeability of the film is a key parameter that determines the compatibility of polymer films for packaging applications. The presence of oxygen is the most common reason for a quality reduction in packaged food. Plasma treatment would break some of C–C/C–H bonds which then are incorporated with plasma generated reactive oxygen species that produce more oxygen‐containing groups on the surface of the film (Pankaj et al., 2015). The mass transfer property is an important element in the determination of the oxygen transfer rate. Crosslinking after plasma treatment is one of the attributes preventing oxygen penetration. The increase of the cross‐linkage between the polymer chains decreases the free volume between them. Therefore, it can be said that the penetration of oxygen into the polymer is thereby reduced (Moosavi et al., 2019).

Several studies have also suggested that intermolecular and interchain linkages and interconnections between radicals and functional groups, present at the plasma‐induced surface, improve the oxygen‐retention properties of the film (Le Tien et al., 2000; Moosavi et al., 2019).

Oxygen barrier properties were improved after plasma treatments (especially with oxygen and argon) due to the increased hydrophobic groups on the film surface. Therefore, high oxygen‐blocking ability plays an important role in maintaining the quality of the packaged product (Hong & Krochta, 2006).

### 3.4 Contact angle

The contact angle test was performed to assess the surface hydrophobicity of the emulsion films. The results are reported in Table 2. The contact angle is an important variable showing how wet a surface is, by measuring the tendency of the liquid droplets to spread on the surface of the solid (Dong et al., 2018). Gelatin is made up of single and double chains that start from hydrophilic heads, forming a three‐dimensional hydrated matrix called hydrogels (Pankaj et al., 2015). Adding oil to the solution and producing an emulsion film slightly increased the contact angle of the control sample. Results showed that contact angle decreased and thus surface hydrophilicity of the GEFs after cold plasma treatment increased (*p* < .05), owing to the increased polar groups and surface roughness. Plasma created radical species at the surface of the polymer which could further be combined with the air oxygen and increased the number of surface polar groups including –OH, C = O, and COOH, in turn increasing the surface hydrophilicity and decreasing the contact angle. O/C ratio at the surface of the film is increased after plasma treatment due to the increase of the concentration of polar groups like C = O and C–O–H, which is the reason for increased hydrophilicity of the gelatin film and increased polar component of the surface free energy (Pankaj et al., 2015).

### 3.5 Thermal analysis

To determine the thermal properties of the GEFs, DSC thermography was performed. Two heat factors including melting temperature (*T*_m_) and melting enthalpy (Δ*H*_m)_ were calculated according to the maximum endothermic peaks of the graphs and compared for different films (Table 3). As can be seen from the thermographs, plasma improved GEFs stability. By applying air gas, melting point increased from 51.9 to 58.9. DBD plasma was responsible for the increase in *T*_m_ and Δ*H*_m_, which can be attributed to changes in the chain motility in the film matrix. The first stage of mass loss (*T*_m onset_) in the control sample was 31.7°C; however, after plasma treatment, a significant difference was observed in the mass loss time of the samples, which increased to 34.5°C in the oxygen‐treated sample. Plasma caused etching effects on the polymer surface (Pankaj et al., 2014). The changes in thermal properties also confirmed that some moisture loss had occurred at the surface of the film (Romani et al., 2019), probably responsible for the modification of *T*_m_ and Δ*H*, because water as a plasticizer would increase the mobility of the chains. Some researchers (Oh et al., 2016; Perez‐Gago & Krochta, 2001) have pointed out that plasma generates radicals at the film surface, affecting film permeability by producing cross‐sections on the film surface. By applying plasma, except for argon gas, an increase in the *T*_m offset_ was observed compared with the control sample. Higher *T*_m offset_ means higher heat capacity and oxidation temperature of the film. By putting these results together, it can be concluded that plasma can modify the thermal properties of films and by applying different types of gases, different results can be achieved.

**Table 3:** Thermal properties of GEFs treated by cold plasma

Plasma gases	*T*_m onset_ (ºC)	*T*_m_ (ºC)	*T*_m offset_ (ºC)	Δ*H*_m_(J/g)
Air	21.9 ± 0.08 ^c^	58.9 ± 0.1^a^	123.8 ± 0.1^b^	421.9 ± 0.02^a^
O_2_	34.5 ± 0.07^a^	57.3 ± 0.2^b^	125.4 ± 0.03^a^	199.0 ± 0.03^c^
N_2_	31.1 ± 0.08 ^b^	56.8 ± 0.1^c^	104.8 ± 0.01^c^	232.8 ± 0.01^b^
Ar	31.5 ± 0.05^b^	51.9 ± 0.3^d^	65.5 ± 0.04^d^	33.0 ± 0.03^e^
EtOH‐Ar	31.1 ± 0.07^b^	56.8 ± 0.1^c^	104.8 ± 0.1^c^	232.8 ± 0.02^b^
Control	31.7 ± 0.05^b^	51.9 ± 0.1^d^	65.5 ± 0.04^d^	34.2 ± 0.01^d^

Note

Data are reported as mean ± *SD*. Means with superscripts of different letters are significantly different (*p* < .05).

Abbreviation: GEFs, gelatin‐based emulsion films.

### 3.6 Surface roughness

**Figure 3:**
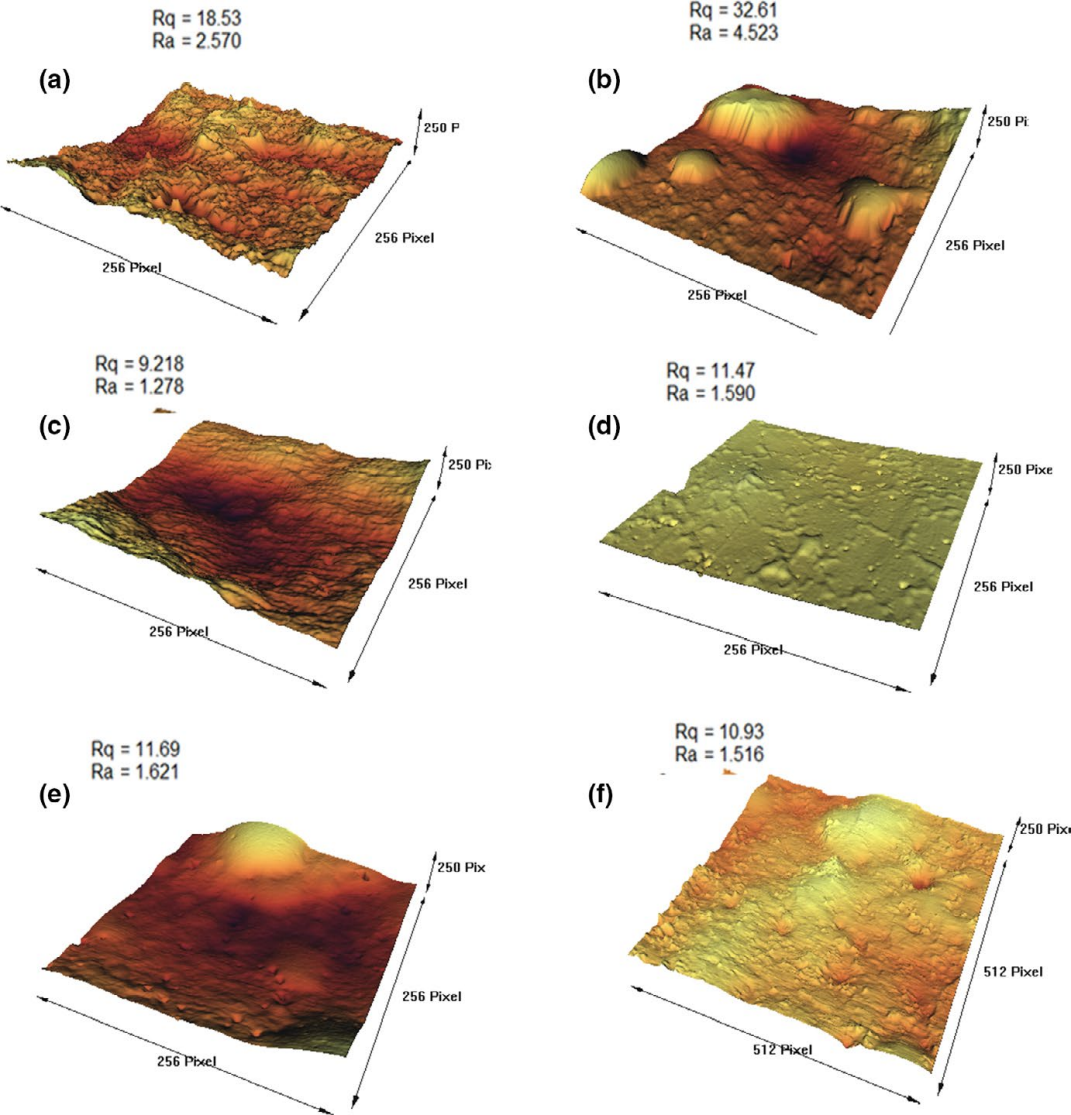
AFM images of surface roughness of cold plasma treated with different gases and untreated GEFs: (a) Air, (b) Ar, (c) EtOH & Ar, (d) O_2_, (e) N_2_, (f) Control. AFM, Atomic force microscopy; GEFs, gelatin‐based emulsion films

To analyze the morphology changes of the film surface after plasma treatment, AFM observations were used to provide a three‐dimensional view. The AFM surface topography and roughness parameters are demonstrated in Figure 3. Two common parameters of average roughness (Ra) and the standard deviation of the evaluated profile (Rq) were used to measure the roughness. The control film exhibited a relatively smooth surface with an average roughness of 1.516 nm. Ra values increased in all samples after plasma treatment, except in the plasma‐treated sample with ethanol‐argon gas, although Rq values increased in the plasma‐treated films using oxygen, nitrogen, argon, and air compared with the control sample. The results showed that plasma treatment modified the surface morphology and significantly increased the surface roughness of the GEFs. As the surface roughness of the films increased, their inhibitory properties against gas permeation increased (Oleyaei et al., 2016). The increase in surface roughness after plasma treatment is related to the effect of bombarding high‐energy plasma species such as ions, electrons, radicals, and ultraviolet radiation on the film surface (Dong et al., 2018). Plasma etching effects would also increase the surface roughness of the films. When the plasma was applied, the energy was transmitted from the plasma to the surface of the film by light irradiation as well as the flux of particles and neutral ions. Bombarding the surface of the film with these energetic particles leads to physical sputtering corrosion (Liu et al., 2004). Moreover, plasma‐activated particles interact with the molecules at the surface resulting in chemical corrosion. During the early stage of plasma discharge, the difference between the sputtering rate of crystal and amorphous structure in films led to a slight accidental morphology. The removed materials by sputtering were decomposed into gas and then diffuse back to the surface. Thus, reaccumulation reactions occurred simultaneous with the corrosion and generated a bumpy surface with a larger specific surface area, therefore, improved the adhesion and wettability (Dong et al., 2018). The plasma etching effect has so far been used to functionalize and clean the surface. Moreover, it can significantly control the release kinetics of active compounds from active packaging materials (Pankaj et al., 2014).

The reduction of surface roughness in ethanol‐argon‐treated samples may be attributed to the shadowing effects. In this theory, reflecting and activating plasma particles have low velocity at the polymer surface grooves, so they can block these grooves and thereby reduce the surface roughness of the film (Moosavi et al., 2019).

### 3.7 Fourier transform infrared spectroscopy

**Figure 4:**
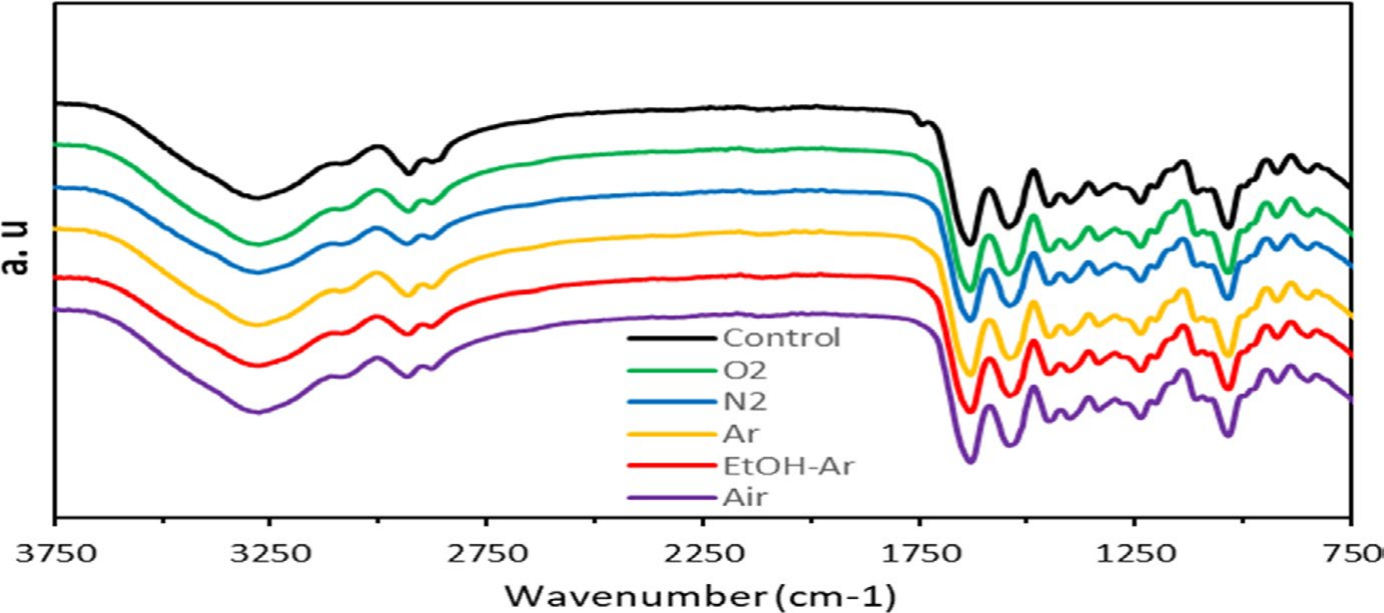
FTIR spectra of cold plasma treated with different gases and untreated GEFs. FTIR, Fourier transform infrared spectroscopy; GEFs, gelatin‐based emulsion films

Fourier transform infrared spectroscopy can be used to study the chemical components and microstructure of GEFs as well as plasma interaction with the film matrix. As shown in Figure 4, the area between 3,200 and 3,500 cm^−1^ was assigned to the amid A region, which might be associated with the O–H and N–H stretch vibration of the hydrogen bonds between the gelatin chains (Rubentheren et al., 2015). The plasma‐treated samples showed deeper adsorption peak with argon gas in this region, attributed to the surface polar groups enrichment, while there were no obvious differences between the four spectra for ethanol‐argon, nitrogen, oxygen, and air treatments. The spectrum between 1,480 and 1,200 cm^−1^ was considered as the fingerprint region for a protein, ascribed to single‐band vibration (C–H, N–H), and amide structure tautomers, respectively. In all film spectra, a peak was observed at approximately 2,930 cm^−1^, which might be related to the amide‐B bonds of the NH_3_^+^ and CH asymmetric reaction (Shankar et al., 2015). The absorption peak at about 1,630 cm^−1^ was related to amide‐I associated with tensile vibration of C = O and amorphous random coil peptides and the peak around 1,540 cm^−1^ corresponded to amide‐II caused by N–H bending and C–N tensile vibration (Andreuccetti et al., 2009).

The amide band III was seen at 1,450 and 1,235 cm^−1^ levels, indicating a C–N and N–H expansion of the surface curvature of the amide bonds and vibrations due to the CH_2_ and CH_3_ groups. In general, the group I of amide represented the secondary structure of the protein, while group II of amide represented the environment for hydrogen bonding (Dong et al., 2018). The band I of amide (1,600–1,700 cm^−1^) was the most prominent and most sensitive vibrational band of the main protein structure. In this region, the bond at about 1,650 cm^−1^ was assigned to α‐helical and disordered compounds (tensile vibration of the C = O bond of amide) and the bond around 1,680 cm^−1^ corresponded to β‐sheets (Singh et al., 2009).

A broad peak was observed at about 1,630 cm^−1^, inconsistent with the α‐helical structure. The increased intensity of this group after DBD plasma treatment showed that plasma treatment increased α‐helical and irregular compounds. An increase in the intensity of the amide‐II band at 1,540 cm^−1^ (bending vibration N–H) was also observed after DBD plasma treatment (Pankaj et al., 2015). This could be justified considering the following reasons: first, wider hydrogen bond between the proteins resulted in a fewer number of nonbonded peptide groups. Second, a portion of β‐sheet structures (the peak at about 1,440 cm^−1^) were deformed into an α‐helical structure. The bands at the range 2,800–3,000, 1,440, and 840 cm^−1^ were also related to hydrocarbon chains (Shi et al., 2010). However, no significant differences were observed in peaks after plasma treatment, indicating that DBD plasma treatment mainly changed surface chemical properties rather than the bulk chemical properties of the films.

### 3.8 Antimicrobial activity

**Figure 5:**
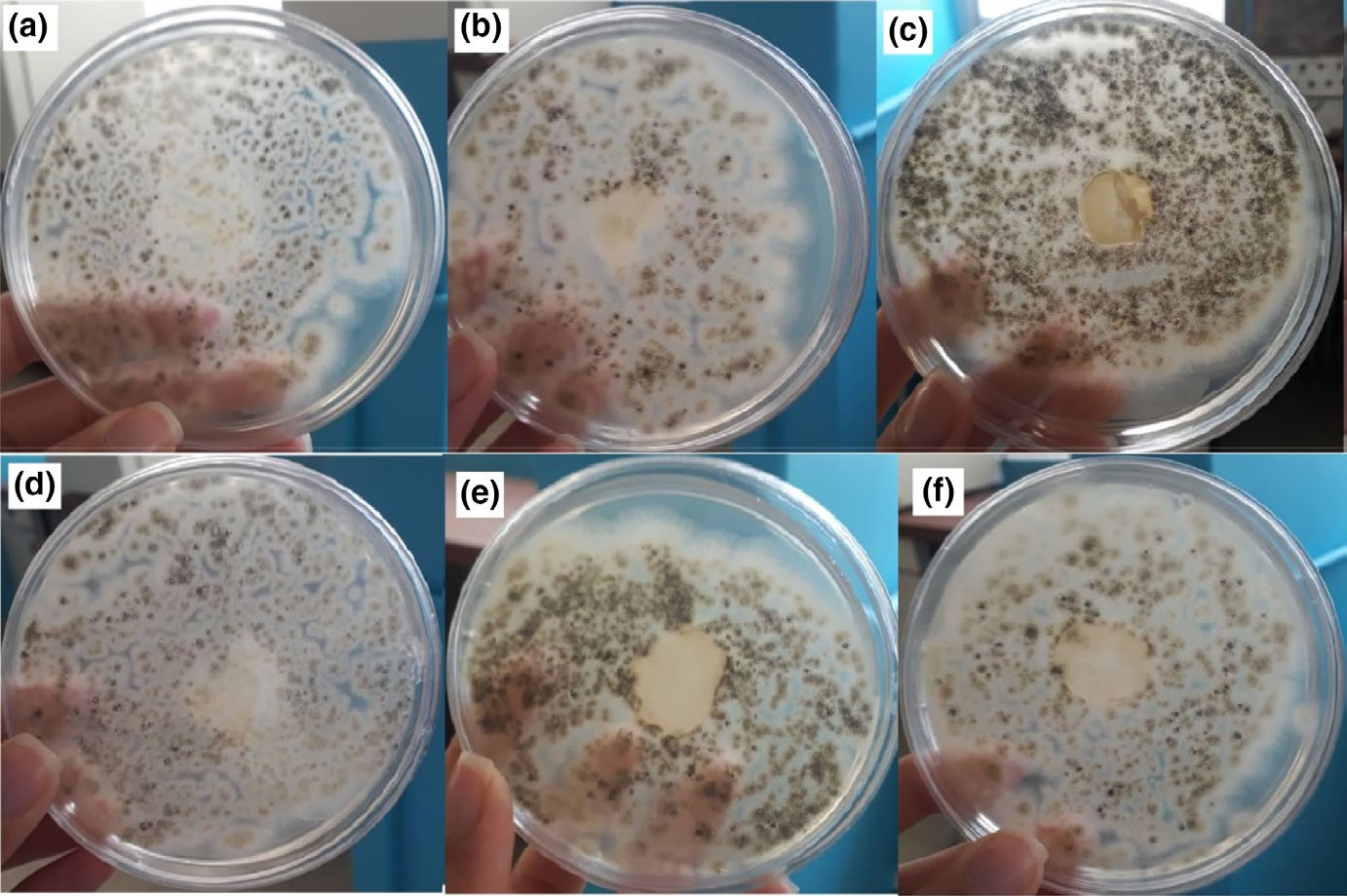
Disk diffusion plates containing *Aspergillusniger* of GEFs: (a) control, (b) EtOH‐Ar, (c) nitrogen, (d) Oxygen, (e) Ar, (f) air after 2 days of incubation at 25°C. GEFs, gelatin‐based emulsion films

To study the effect of plasma on the antimicrobial properties of the GEFs, the inhibition area of the spherical film fragments was measured (Figure 5). As shown, the plasma‐treated films showed better antimicrobial activity compared with the control sample. Most microbial inhibition is related to air plasma gases and nitrogen, probably due to the active groups of amines capable of binding to anionic groups at the cell wall surface and disrupting the activity of the microbial species. The area of inhibition of the film fragments was measured with a digital caliper; the results of which are shown most inhibition are related to air and nitrogen gas (retention area 200 mm^2^). In addition to functional oxygen‐containing groups, air plasma gas could also introduce amines and amide groups owing to the air nitrogen. Plasma treatment with nitrogen gas also forms amide bonds on the surface of the polymer. But, as can be seen in Figure 5, no antifungal activity was observed in untreated plasma samples.

## 4 CONCLUSION

Results proposed that cold plasma treatment can effectively improve the wettability of GEFs. The thermal properties of the GEFs increased after plasma treatment in air and oxygen. The plasma‐treated films showed antimicrobial activity, with the highest area of antimicrobial inhibition related to air and nitrogen gases. Plasma treatment significantly reduced oxygen permeability compared with the control sample. The greatest decrease in oxygen permeability was observed in plasma oxygen‐treated samples. The contact angle of the GEFs was reduced after plasma treatment. The most significant decrease in contact angle was in the plasma‐treated sample with ethanol‐argon gas. Cold plasma treatment is an effective method for surface modification, expanding the application of emulsion films in the packing industry with improved performance properties. The safety analysis of edible films treated with cold plasma can be a substantial part of future studies to prevent the use of unwanted compounds, adversely affecting the safety of films and human health.

## Data Availability

The data that support the findings of this study are available from the corresponding author upon reasonable request.
